# From Data Poverty to Data Sovereignty: Operationalizing Gold-Standard Biomedical Datasets in Low- and Middle-Income Countries

**DOI:** 10.2196/82360

**Published:** 2026-08-10

**Authors:** Shweta Rana, Pranshu Bhagat, Debnath Pal, Sanghamitra Pati, Harpreet Singh

**Affiliations:** 1 Division of Development Research, Indian Council of Medical Research, Ansari Nagar, Delhi, New Delhi, India, 91 9999496965; 2 Corporate Strategy & Quality (CEO's office), Piramal Group & Strategy, Mumbai, Maharashtra, India; 3 Wadhwani Institute of Artificial Intelligence, New Delhi, New Delhi, India; 4 Department of Computational and Data Sciences, Indian Institute of Science (IISc), Bangalore, Karnataka, India; 5 Indian Council of Medical Research, Delhi, New Delhi, India; 6 Faculty of Medical Sciences, Academy of Scientific and Innovative Research, Ghaziabad, Uttar Pradesh, India

**Keywords:** data poverty, data sovereignty, gold standard, low- and middle-income country, LMIC, biomedical datasets

## Abstract

AI is rapidly expanding in health care. However, there is a significant underrepresentation of low- and middle-income countries (LMICs) in datasets used to train AI applications. Current Food and Drug Administration (FDA)–approved AI tools predominantly use data from high-income countries, with fewer than 4% reporting geographic or racial diversity. This geographic skew leads to significant performance degradation when these tools are used in LMIC populations, perpetuating an equity crisis where health burdens are highest.

Addressing this disparity, we highlight the Medical Imaging Datasets for India (MIDAS) initiative as a viable model to transition LMICs from “data poverty” to “data sovereignty.” MIDAS uses a rigorous, 4-domain Dataset Quality Matrix to ensure representativeness, documentation, technical fidelity, and governance, thereby creating openly benchmarked, gold-standard datasets tailored to local contexts. Initial releases, including datasets for oral and dural lesions, demonstrate the feasibility and practical value of developing robust, generalizable AI models.

Furthermore, we propose a multilateral South-South Data Commons structured around 3 foundational pillars: a harmonized dataset-grading rubric, distributed custodial governance, and outcome-linked incentives. This infrastructure supports local stewardship, encourages global collaboration, and ensures that quality benchmarks drive financial incentives for dataset expansion and diversity.

This proposed framework not only positions LMICs as autonomous data stewards but also enhances global AI equity. By institutionalizing quality control, interoperability, and outcome accountability, LMICs can transform from passive data consumers into active contributors to essential, trustworthy, and globally relevant biomedical datasets.

## Background

The last decade has seen an explosion of AI tools approved for clinical use, yet fewer than 4% of the studies submitted to the US Food and Drug Administration (FDA) reported any racial or geographic diversity, and almost none disclosed socioeconomic data [1]. Systematic assessments show that the publicly accessible training corpora for clinical AI are drawn disproportionately from a small cluster of wealthy nations, leaving most of the world essentially invisible in the data landscape. Among 7314 PubMed-indexed clinical AI studies analyzed in a 2022 global review, 71% of the datasets originated in just 10 high-income countries (HICs), with the United States alone contributing 41%. This underscores the pronounced geographic skew that shapes model development [2]. Predictably, the devices trained on data from HIC cohorts have shown unstable performance when deployed in low- and middle-income country (LMIC) settings, undermining confidence in AI as an enabler of universal health coverage [3]. The resulting “health-data poverty” [4] is no longer merely a research inconvenience; it is an equity crisis that threatens to widen outcome gaps precisely where disease burdens are greatest [5].

In this paper, we outline how LMIC-led, quality-graded, openly benchmarked data frameworks, exemplified by the Medical Imaging Datasets for India (MIDAS) program, can provide a practical solution for transitioning from data poverty to data sovereignty. Data sovereignty refers to institutional decision-making authority and accountability over the full data lifecycle (including collection, curation, access terms, downstream validation, and benefit-sharing) exercised by the institutions closest to the data subjects. It is distinct from data localization, which is primarily a jurisdictional requirement governing where data are stored or processed, and from data access, which concerns permissions to use data without necessarily shifting custodianship or governance authority. By enabling locally governed, demographically annotated datasets, this approach also supports equity for historically marginalized populations within LMICs, including rural communities, ethnic minority groups, and socioeconomically disadvantaged groups whose clinical representation is systematically absent from global AI benchmarks. We also propose a multilateral South-South Data Commons built on 3 pillars: a harmonized dataset-grading rubric, distributed custodial governance, and outcome-linked incentives for AI-enabled products.

## A Landscape Defined by Opaqueness and Asymmetry

Commercial enthusiasm has yielded more than 1000 FDA-listed AI-enabled devices, yet transparency gaps remain striking. Only 3.6% of regulatory dossiers disclose race or ethnicity, and fewer than 1% report socioeconomic indicators. Among the regulatory dossiers, 46.1% provided detailed performance data, 1.9% provided a link to a scientific publication, and only 9% included a prospective study for postmarket surveillance [1]. Similar deficits plague public benchmark datasets: a systematic review in *The Lancet Digital Health* identified major documentation shortfalls in more than 75% of the COVID-19 pandemic datasets most frequently reused for AI development [6].

Although radiology societies advocate for radically open corpora, the public availability of population-level imaging datasets remains scarce, and nearly all such datasets originate from high-income settings. Many studies have highlighted this geographical skew in datasets. Using the MEDLINE database, Google’s search engine, and Google Dataset Search, a set of 94 open access datasets containing 507,724 images and 125 videos from 122,364 patients were identified. Most datasets originated from Asia, North America, and Europe [7]. A 2023 review of 110 magnetic resonance imaging (MRI) datasets found no datasets from LMICs, underscoring how this geographic skew hampers the development of robust, generalizable AI models for global use [8]. The resulting geographic skew is now well cataloged: models trained on US and EU chest radiograph data lose up to 25% points in the area under the curve when validated in African populations, even after standard transfer learning [9]. The WHO 2024 guidance on AI governance warns that nonrepresentative datasets can entrench structural inequities at scale and urges member states to cultivate AI learning ecosystems that advance equity and safety [10].

## Data Sovereignty: Beyond Access to Agency

The current solutions for providing LMICs with access to datasets from HICs treat the problem as a simple data shortage rather than as an issue of unequal power. The concept of data sovereignty reframes the debate: the origin of the data determines both their scientific reliability and the authority over their use. A *BMJ Global Health* scoping review shows that local stewardship improves adherence to context-specific ethical norms and accelerates regulatory approvals [11]. Sovereignty, however, demands more than national firewalls; it requires interoperable standards that enable cross-border collaboration without ceding custodial control. In this manuscript, data sovereignty is not treated as synonymous with data localization or access restriction, but as institutional agency across the AI development, validation, and deployment lifecycle. Table 1 summarizes the differences between the prevailing HIC-centric AI development pipeline and the proposed LMIC data sovereignty model across key stages of the AI lifecycle. One concrete step in this direction is the MIDAS platform, which is a collaborative effort between the Indian Council of Medical Research (ICMR), the Indian Institute of Science (IISc), and AI and Robotics Technology Park (ARTPARK) to develop high-quality, standardized, and diverse datasets for developing contextually relevant, AI-driven health care solutions in India. Using a hub-and-spoke model, MIDAS enables the systematic collection, annotation, and harmonization of multimodal clinical data.

**Table 1.** Comparison of the current high-income country (HIC)–centric AI development pipeline and the proposed low- and middle-income country (LMIC) data sovereignty model.^a^

Steps in the AI pipeline	Current HIC-centric model	Proposed LMIC data sovereignty model
Dataset origin	Data sourced from HICs (eg, United States and Europe)	Data sourced from local LMIC institutions through MIDAS^b^ or similar frameworks
Data quality oversight	Minimal or nontransparent grading; dataset quality varies	Dataset quality graded using structured frameworks (eg, MIDAS Quality Matrix)
Demographic representation	Often lacks racial, geographic, and socioeconomic diversity	Designed to capture local population diversity (age, gender, ethnicity, and geography)
Model development	Trained on HIC datasets, often generalized for global use	Trained and validated on representative LMIC datasets
Evaluation and audit	Performance metrics reported by developers, often lacking external validation	External, privacy-preserving audits with third-party evaluators
Regulatory pathway	US Food and Drug Administration approval or *Conformité Européenne* marking based on HIC data; limited LMIC relevance	Contextualized validation mechanisms with LMIC participation
Deployment and use	Deployed globally without local adaptation; performance issues common in LMICs	Deployed with outcome-linked incentives tied to local performance and equity metrics

a This table contrasts the prevailing HIC-driven AI development pathway with a proposed LMIC-led framework grounded in data sovereignty principles. The proposed framework emphasizes quality grading (eg, Medical Imaging Datasets for India), local custodianship, demographic diversity, and incentivized deployment through validated benchmarks.

b MIDAS: Medical Imaging Datasets for India.

To operationalize these principles, MIDAS uses a 4-domain Dataset Quality Matrix covering representativeness, documentation, technical fidelity, and governance. Scores (0‐100) map onto Bronze, Silver, Gold, Platinum, and Diamond grades, with an external audit conducted prior to public release (Multimedia Appendix 1). The first gold-graded corpus, comprising 1000 oral-lesion images drawn from 6 Indian cancer centers, was released in October 2024 under a Creative Commons Attribution–NonCommercial (CC-BY-NC) license [12]. Recently, the dataset was onboarded onto AIKosh [13], India’s national AI repository. Within 1 month, the dataset ranked third among trending datasets on the platform (out of 5577 datasets) and was downloaded 738 times, indicating early uptake by the AI research and developer community. A Gold-graded dural lesion dataset followed in March 2025, codeveloped with the Department of Neurosurgery, All India Institute of Medical Sciences (AIIMS), New Delhi. Crucially, both datasets are anonymized; however, they include demographic fields such as age, gender, and ethnicity, which are required for developing robust AI applications, as well as versioned consent artifacts, thereby addressing the transparency gaps currently evident in FDA filings.

Global data-sovereignty efforts such as the African Health Data Space and the Organisation for Economic Co-operation and Development (OECD) Health Data Governance Principles have emphasized legal interoperability, ethical safeguards, and cross-border data-sharing norms. These frameworks are critical for establishing trust and harmonization but largely stop short of operationalizing sovereignty within the AI development lifecycle itself. MIDAS complements these initiatives by introducing a quantitative, auditable dataset-grading framework that links local stewardship to model validation, benchmarking, and outcome-linked incentives. Rather than focusing solely on access governance or data flows, MIDAS embeds sovereignty at the level of dataset readiness and evaluative authority, enabling LMIC institutions to influence how AI systems are tested, certified, and rewarded. This positions MIDAS not as an alternative to existing governance frameworks, but as an implementation layer that translates global principles into measurable, AI-relevant practice.

## Toward a South-South Data Commons

**Figure 1.**
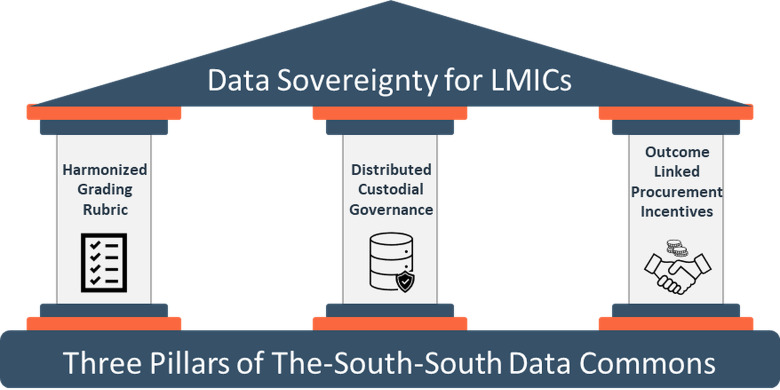
Three pillars of the South-South Data Commons Framework. A self-reinforcing cycle representing how low- and middle-income countries (LMICs) can transition from data poverty to data sovereignty. Local institutions generate graded datasets (eg, via Medical Imaging Datasets for India), which are used by AI developers and externally validated. Models that meet performance thresholds receive outcome-linked incentives, supporting reinvestment in further data curation and strengthening the ecosystem.

Building on the MIDAS foundation, we propose a 3-pillar framework for establishing a South-South Data Commons that transcends bilateral collaborations to create a genuinely multilateral infrastructure (Figure 1).

## Harmonized Grading Rubric

Replicating MIDAS without coordination risks a balkanized landscape of incompatible locally developed metrics. We therefore endorse the adoption of a common, open-source rubric extending MIDAS to nonimaging modalities and aligning it with the emerging Medical AI Data for All (MAIDA) framework for global medical datasets [14]. These perspectives have called for a “shared quality lingua franca” to facilitate cross-registry discovery [15]; a South-South rubric jointly maintained by the ICMR, the IISc, and the African Health Data Space could satisfy that mandate while preserving local autonomy [16].

## Distributed Custodial Governance

Distributed custodial governance within the proposed South-South Data Commons is operationalized through a clear separation of roles across data stewardship, technical validation, and trust certification. Primary custodianship remains with the institutions closest to the data subjects, which retain authority over consent management, ethics approvals, and long-term stewardship of datasets. Technical validation can be distributed and conducted by designated nodal evaluators such as public research institutions or accredited AI evaluation laboratories with domain expertise using predefined grading rubrics such as the MIDAS Quality Matrix. Trust and certification are established through publicly accessible, versioned artifacts, including dataset grades, audit summaries, and metadata records hosted on national platforms such as AIKosh. The Trustworthy Evaluation of Clinical AI consortium has demonstrated that external evaluators can audit diabetic retinopathy algorithms using encrypted uploads without exposing patient-level data [17]. This functional separation allows local institutions to retain sovereignty while enabling credible, repeatable, and scalable validation, thereby preventing centralization and reinforcing mutual accountability within the Commons [17].

## Outcome-Linked Procurement Incentives

**Figure 2.**
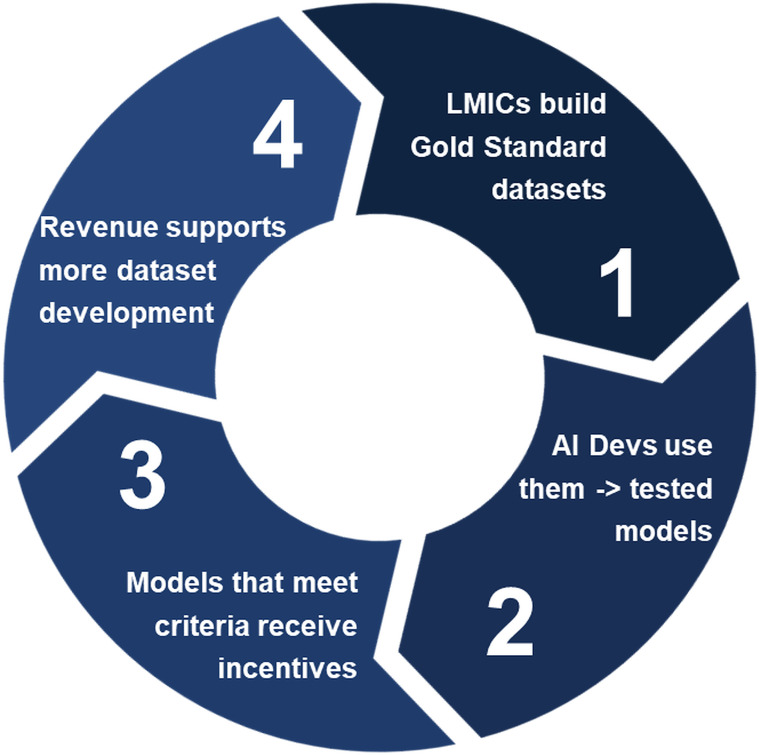
Feedback loop of the low- and middle-income country (LMIC) data sovereignty model. A self-reinforcing cycle representing how LMICs can transition from data poverty to data sovereignty. Local institutions generate graded datasets (eg, via Medical Imaging Datasets for India), which are used by AI developers and externally validated. Models that meet performance thresholds receive outcome-linked incentives, supporting reinvestment in further data curation and strengthening the ecosystem.

Market pull is indispensable. England’s National Health Service (NHS) AI Award releases final-stage funds only when applicants’ algorithms meet accuracy and fairness targets on the test partition of the National COVID-19 Chest Imaging Database [18]. By hard-wiring gold-standard dataset performance into payment schedules, these programs turn data quality into a revenue driver and create a self-reinforcing loop: vendors eager for outcome bonuses invest in expanding and diversifying the very datasets that will be used to evaluate the next procurement round. Governance is operationalized through transparent evaluation criteria, third-party validation, and outcome-linked disbursement rather than discretionary funding, thereby operationalizing sovereignty as participation in value creation rather than passive data provision (Figure 2).

## Anticipated Challenges

### Regulatory Fragmentation

Although many LMICs have enacted data-protection statutes (eg, India’s Digital Personal Data Protection [DPDP] Act 2023), few contain explicit provisions for cross-border clinical-research data flows. Regulators should adopt a “trust framework” akin to the EU-US Data Privacy Framework [19], enabling Commons participants to exchange deidentified data under reciprocal adequacy findings.

### Sustainability

Dataset curation is costly, requiring ongoing investment to maintain quality and support updates [20]. A blended-finance approach, combining World Bank digital health loans, philanthropic grants, and small levies on AI-assisted clinical tests, has been recommended to fund the longitudinal upkeep of biomedical datasets [21]. Such models are increasingly recognized as effective strategies for supporting health care data infrastructure, particularly in LMICs. Programs such as the All of Us Research Program emphasize the importance of sustained curation and highlight its role in enabling downstream research. However, specific quantitative returns on investment have not yet been established in the published literature [22].

### Technical Debt

Version creep and annotation drift loom large. Federated continuous integration pipelines can enforce schema consistency only if maintainers budget for reannotation cycles and retain cloud credits for secure computing. The MIDAS dataset already schedules “maintenance releases,” in which new images are rescored, and governance metadata are refreshed [12].

## Global Implications

Transitioning from data scarcity to sovereignty is more than an LMIC agenda; it is a prerequisite for valid, generalizable AI everywhere. Commercial developers seek regulatory clarity, journal editors demand reproducibility, and patients deserve equity by design. A South-South Commons anchored in graded, openly documented datasets would convert what WHO terms “an ethical imperative” into a tangible, auditable infrastructure. HIC regulators and payers stand to benefit: models validated on Commons data will arrive already stress-tested across demographic gradients that do not exist in single-country registries, reducing the risk of catastrophic postdeployment failures.

## Conclusions

Algorithmic equity cannot be retrofitted after deployment; it must be engineered upstream through the data themselves. By institutionalizing rigorous quality grading, distributed custodianship, and value-linked incentives, LMICs can recast themselves from data supplicants to data sovereigns producing datasets that are not only locally trustworthy but also globally indispensable. The blueprint outlined here, grounded in the MIDAS experience and amplified through a South-South Data Commons, offers a practical, scalable route toward that future.

From a policy perspective, operationalizing data sovereignty does not require a wholesale system redesign. Immediate steps for LMIC governments and research agencies include formally adopting transparent dataset quality grading standards within publicly funded research programs; designating or accrediting national institutions to perform independent, privacy-preserving AI validation; and embedding gold-standard dataset performance requirements into public procurement, regulatory evaluation, and grant funding criteria. National data platforms can be made interoperable with Commons architectures by publishing versioned metadata, audit artifacts, and validation outcomes rather than raw data alone. Taken together, these measures allow sovereignty to be exercised incrementally through evaluative authority, incentive alignment, and regulatory participation while remaining compatible with cross-border collaboration and global AI development norms.

## Supplementary material

10.2196/82360 Multimedia Appendix 1 Four-domain Dataset Quality Matrix.

## Abbreviations

AIIMS: All India Institute of Medical Sciences
ARTPARK: AI and Robotics Technology Park
CC-BY-NC: Creative Commons Attribution-NonCommercial
DPDP: Digital Personal Data Protection
FDA: Food and Drug Administration
HIC: high-income country
ICMR: Indian Council of Medical Research
IISc: Indian Institute of Science
LMIC: low- and middle-income country
MAIDA: Medical AI Data for All
MIDAS: Medical Imaging Datasets for India
MRI: magnetic resonance imaging
NHS: National Health Service
OECD: Organisation for Economic Co-operation and Development
