# Supplementary material for: From Data Poverty to Data Sovereignty: Operationalizing Gold-Standard Biomedical Datasets in Low- and Middle-Income Countries
Source: JMIR Med Inform. 2026 Aug 10;14:e82360. doi: 10.2196/82360 (PMC13456305; doi:10.2196/82360)
Supplement: Supplementary file 1 [file medinform-v14-e82360-s001.docx]

**Four-domain Dataset Quality Matrix**

**1. Documentation**

| **Score** | **Description** |
| --- | --- |
| **0** | No documentation. Only data files exist. External users would not understand columns or measurement units. |
| **1** | A short note or document explains variables but lacks collection methods, consent, or cleaning steps. |
| **2** | Variable definitions, measurement methods, and consent statements included. Users can read and interpret values correctly. |
| **3** | Clear SOPs or manuals, change logs, and limitation notes are attached. Users can replicate data processing confidently. |
| **4** | Complete guide with examples, diagrams, and data-use policy publicly accessible. Allows immediate understanding and reuse by external researchers. |

**2. Representativeness**

| **Score** | **Description** |
| --- | --- |
| **0** | Source population unclear. Data may come from a small convenience sample or single location. |
| **1** | Contains demographic fields (age, sex, location) but from one facility or group. Limited diversity and external validity. |
| **2** | Includes records from multiple sites or regions, covering varied demographics but no balance or gap analysis performed. |
| **3** | Dataset includes a summary table comparing actual vs target enrolment for age, sex, or geography; imbalances documented. |
| **4** | Representativeness periodically reviewed (e.g., quarterly). Under-represented groups flagged and corrections attempted. Provides evidence of true diversity. |

**3. Technical Fidelity**

| **Score** | **Description** |
| --- | --- |
| **0** | Data were entered by a single person without supervision or checking. No evidence that labels, diagnoses, or responses were reviewed for correctness. |
| **1** | Occasional or informal checking by another person, but no written record of who checked or what was corrected. |
| **2** | Two people have reviewed at least some records, discussed differences informally, but without recording results. |
| **3** | Most records reviewed independently by two trained persons; disagreements settled by a senior reviewer. Records of corrections exist. |
| **4** | A well-defined double-review system used for all data. Agreement consistently high (>80%). Correction logs and reviewer names recorded. |

**4. Governance**

| **Score** | **Description** |
| --- | --- |
| **0** | No named custodian or data-protection oversight. Ownership unclear; dataset unmanaged after creation. |
| **1** | Custodian identified but responsibilities undefined. Governance relies on individual rather than system. High continuity risk if personnel change. |
| **2** | Data-processing register (what, why, where stored) exists. Data-protection impact assessment completed. Compliance documentation traceable. |
| **3** | Governance reviewed periodically; metrics such as access requests and incident logs tracked. |
| **4** | Independent audit evaluated governance framework; deficiencies corrected. Public statement or report shared. Dataset demonstrates accountability with data-protection law. |

**How the Scores are Calculated**

Each dataset is assessed across **4 domains**, each scored from **0 to 4**, where *0 = absent* and *4 = exemplary*.

The **Composite Quality Index -Lite (CQI)** is computed as:

$$CQI=\frac{\text{Sum of domain scores}}{\text{Maximum possible score}}\times100$$

Use the highest level where all statements are true. If any statement at that level is missing, step down one level.

**How the Scores are Interpreted**

The **Composite Quality Index-Lite (CQI)** determines the overall dataset quality, classified into six performance bands as follows:

| **Aggregated CQI band** | **Grade** | **Interpretation** |
| --- | --- | --- |
| ≥ 95 | Diamond | Global exemplar |
| 85 – 94 | Platinum | Best‑practice dataset |
| 70 – 84 | Gold | High‑quality dataset |
| 50 – 69 | Silver | Permissible but improvement plan must be recorded |
| 25 – 49 | Bronze | Embargo until targeted enhancements completed |
| < 25 | Remediation | Iterative QA and resubmission required |
